# Isorhamnetin Ameliorates Dry Eye Disease via CFTR Activation in Mice

**DOI:** 10.3390/ijms22083954

**Published:** 2021-04-12

**Authors:** Ho K. Lee, Jinhong Park, Bo-Rahm Kim, Ikhyun Jun, Tae-im Kim, Wan Namkung

**Affiliations:** 1 College of Pharmacy and Yonsei Institute of Pharmaceutical Sciences, Yonsei University, 85 Songdogwahak-ro, Yeonsu-gu, Incheon 21983, Korea; jason.hoking@gmail.com (H.K.L.); jjinung@yonsei.ac.kr (J.P.); 2 Interdisciplinary Program of Integrated OMICS for Biomedical Science Graduate School, Yonsei University, Seoul 03722, Korea; 3 Department of Ophthalmology, College of Medicine, Yonsei University, 50 Yonsei-ro, Seodaemoon-Gu, Seoul 03722, Korea; cjlovem@naver.com (B.-R.K.); HADESDUAL@yuhs.ac (I.J.); TIKIM@yuhs.ac (T.-i.K.)

**Keywords:** isorhamnetin, cystic fibrosis transmembrane conductance regulator, dry eye

## Abstract

Dry eye disease is one of the most common diseases, with increasing prevalence in many countries, but treatment options are limited. Cystic fibrosis transmembrane conductance regulator (CFTR) is a major ion channel that facilitates fluid secretion in ocular surface epithelium and is a potential target of therapeutic agent for the treatment of dry eye disease. In this study, we performed a cell-based, high-throughput screening for the identification of novel natural products that activate CFTR and restore the aqueous deficiency in dry eye. Screening of 1000 natural products revealed isorhamnetin, a flavonol aglycone, as a novel CFTR activator. Electrophysiological studies showed that isorhamnetin significantly increased CFTR chloride current, both wild type and ∆F508-CFTR. Isorhamnetin did not alter intracellular cAMP levels and the activity of other ion channels, including ANO1, ENaC, and hERG. Notably, application of isorhamnetin on mouse ocular surface induced CFTR activation and increased tear volume. In addition, isorhamnetin significantly reduced ocular surface damage and expression of interleukin (IL)-1β, IL-8, and tumor necrosis factor (TNF)-α in an experimental mouse model of dry eye. These data suggest that isorhamnetin may be used to treat dry eye disease.

## 1. Introduction

Dry eye disease is a common ocular disease with a broad group of ocular conditions that are caused by inadequate production of tears and excessive tear evaporation, resulting in lack of ocular lubrication [1]. Dry eye disease is characterized by symptoms of dryness, pressure behind the eye, and other inflammation-related symptoms that affect quality of life [2,3]. Approximately 5~54.3% of the population suffers from the disease, making it one of the most prevalent diseases in the world [4]. Recent studies on dry eye disease have shown that inflammation of the lacrimal gland, meibomian gland, cornea, and conjunctiva plays an important role in its pathogenesis, with a marked increase in tear inflammatory cytokines and immune cell infiltration [5].

Currently available treatments for the dry eye disease include artificial eye drops, which can moisten the eyes for a short period of time upon treatment before it evaporates, and cyclosporine, which is used to subdue inflammation [6,7]. However, these treatments only give temporary relief by indirectly suppressing symptoms, and there are no treatments available that can last longer while moisturizing the eye. Recently, P2Y_2_ receptor agonists, such as diquafosol, which could restore the tear film stability by transiently increasing water secretion from calcium-activated chloride channels expressed on the conjunctival epithelium, have been suggested as a possible treatment for the disease [8]. Various ion channels including cystic fibrosis transmembrane conductance regulator (CFTR), anoctamin 1(ANO1), and ENaC have been identified in the conjunctival epithelium, which produces mucin and tear, and the ion channels play pivotal roles in the regulation of tear film homeostasis [9,10,11]. CFTR is a cAMP-regulated chloride channel expressed in various epithelia including airway, intestinal, and ocular epithelia. Mutations in CFTR, which impair its expression and function, cause cystic fibrosis (CF), the most common lethal genetic disease, and CF patients showed that they exhibit ocular surface abnormality of low tear film stability [12].

Recent studies showed that forskolin-induced activation of CFTR was detected in mouse, rat, rabbit, and human ocular epithelium and revealed CFTR is a promising target for a new therapeutic agent for the treatment of dry eye disease [11]. In human ocular surface, increasing the fluid transport via CFTR activators can induce sustained flow of water compared to the transient activation of calcium-activated chloride channels with the P2Y_2_ agonist [13], and these results strongly suggest that CFTR activators are potential agents for the treatment of dry eye. The fluid secretion from the ocular epithelia could restore the impaired aqueous tear film layer of the dry eye patients. In this study, we identified a novel CFTR activator, isorhamnetin, and investigated the effectiveness of isorhamnetin in an experimental mouse model of dry eye.

## 2. Results

### 2.1. Identification of CFTR Activator

**Figure 1:**
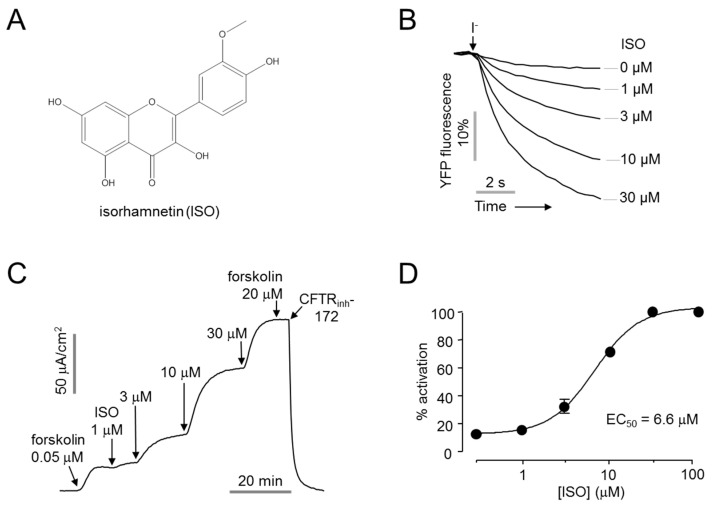
Identification of novel CFTR activator, isorhamnetin. (**A**) Chemical structure of isorhamnetin (**B**) YFP fluorescence measured in CHO-K1 cells expressing CFTR and YFP-F46L/H148Q/I152L. Indicated concentrations of isorhamnetin were applied 10 min prior to extracellular I^-^ addition. (**C**) Apical membrane current measured in WT-CFTR expressing FRT cells. CFTR was activated by indicated concentrations of isorhamnetin and forskolin, CFTR agonist, and inhibited by 10 µM CFTR_inh_-172. (**D**) Dose-response curve (mean ± S.E., *n* = 3–4).

A cell-based screening of 1000 natural products was performed for the identification of novel CFTR activators. The effect of the natural products on CFTR activity was measured with YFP quenching assay in CHO-K1 cells stably expressing human CFTR and a halide sensor YFP-F46L/H148Q/I152L. The screening revealed isorhamnetin, which significantly increased CFTR chloride channel activity in a dose-dependent manner (Figure 1A,B). Apical membrane currents were measured to verify the effect of isorhamnetin on CFTR chloride channel activity in WT-CFTR expressing FRT cells. Isorhamnetin fully activated CFTR at 30 μM with EC_50_ of 6.6 ± 1.1 µM, and CFTR chloride current was not further increased by 20 μM forskolin, inducing maximal activation of CFTR (Figure 1C,D).

### 2.2. Characterization of Isorhamnetin

**Figure 2:**
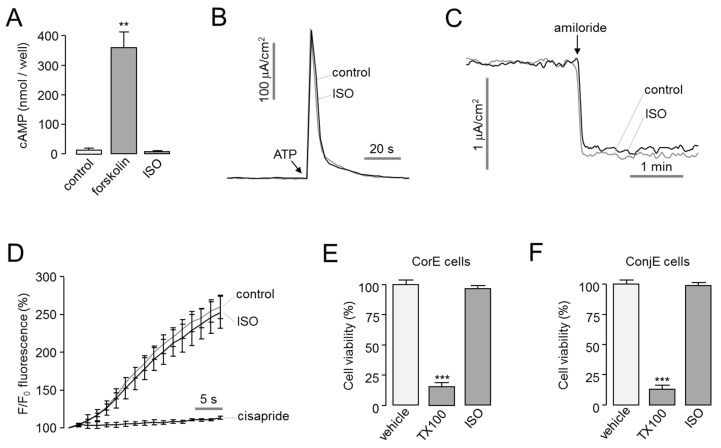
Characterization of isorhamnetin. (**A**) cAMP accumulation in FRT cells in response to addition of isorhamnetin (30 µM) and forskolin (20 µM) (mean ± S.E., *n* = 3–4). (**B**) Apical membrane currents were measured in FRT-ANO1 cells. Isorhamnetin 30 μM was added 10 min prior to ANO1 activation by 100 μM ATP. (**C**) Effect of isorhamnetin on ENaC activity was observed in T84 cells. Isorhamnetin 30 μM was applied 10 min prior to ENaC inhibition by 100 µM amiloride. (**D**) Effect of isorhamnetin on hERG potassium channel activity was measured in HEK293T cells expressing hERG (mean ± S.E., *n* = 3). Isorhamnetin 30 μM was pretreated for 10 min. The hERG channel was inhibited by 50 μM cisapride. (**E**,**F**) CorE and ConjE cells were treated with isorhamnetin (30 µM) for 48 h and cell viability was determined by MTS assay. Triton X-100 (TX100, 0.01%) was used as a positive control (mean ± S.E., *n* = 4–6). ** *p* < 0.01, *** *p* < 0.001.

To investigate the effect of isorhamnetin on the intracellular cAMP signaling, which induces CFTR activation, cAMP concentration changes by forskolin, an adenylyl cyclase activator, and isorhamnetin were observed in FRT cells. Isorhamnetin did not alter the intracellular cAMP levels (Figure 2A). In previous study, the effects of a small-molecule CFTR activator on other ion channels affecting tear homeostasis were investigated [14]. So, we observed the effect of isorhamnetin on ANO1 and ENaC channels expressing in ocular epithelium. Apical membrane current of ANO1 was measured in FRT cells expressing human ANO1, and ENaC short-circuit current was measured in T84 cells expressing ENaC. Isorhamnetin did not alter ANO1 and ENaC function (Figure 2B,C). To investigate the cardiac safety assessment of isorhamnetin, the effect of isorhamnetin on hERG potassium channel activity was observed in HEK293T cells expressing hERG by FluxOR potassium ion channel assay. Isorhamnetin did not affect hERG channel activity (Figure 2D). To determine the cytotoxic effect of isorhamnetin, cell viability was measured using MTS assay in corneal epithelial (CorE) cells and conjunctival epithelial (ConjE) cells. In both CorE and ConjE cells, isorhamnetin did not affect the cell viability at 30 μM and Triton X-100 (0.01%) markedly decreased cell viability (Figure 2E,F).

### 2.3. Whole-Cell Patch Clamp in CHO-CFTR Cells

**Figure 3:**
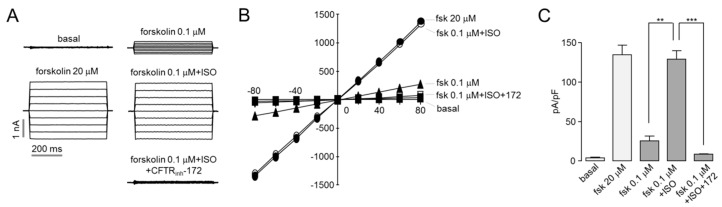
Isorhamnetin fully activates CFTR chloride currents. (**A**) Whole-cell currents were recorded at a holding potential at 0 mV, and pulsing to voltages between ± 80 mV (in steps of 20 mV) in CHO-K1 cells expressing WT-CFTR. CFTR was activated by indicated concentrations of forskolin and isorhamnetin and inhibited by 10 μM CFTR_inh_-172. (**B**) Current/voltage plot of mean currents at the middle of each voltage pulse. (**C**) Summary of current density data measured at +80 mV (mean ± S.E., *n* = 3–5). ** *p* < 0.01, *** *p* < 0.001.

To further characterize activation of CFTR by isorhamnetin, whole-cell recording performed on CHO-K1 cells expressing CFTR. As shown in Figure 3, application of 30 μM isorhamnetin fully activated CFTR Cl^-^ currents exhibit a linear current/voltage relationship. The isorhamnetin-induced CFTR Cl^-^ currents were almost completely blocked by CFTR_inh_-172.

### 2.4. Isorhamnetin Potentiates Low-Temperature-Rescued ΔF508-CFTR

**Figure 4:**
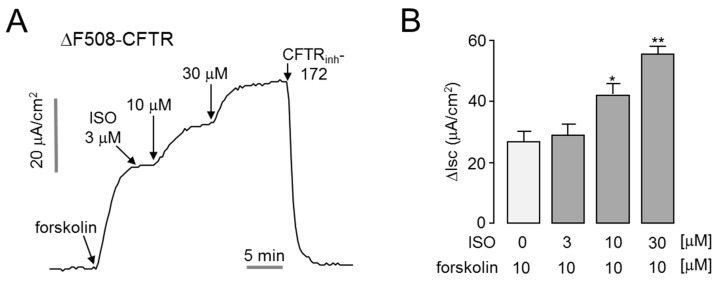
Isorhamnetin potentiates low-temperature-rescued ΔF508-CFTR. (**A**) Apical membrane current was measured in FRT cells expressing human ΔF508-CFTR. ΔF508-CFTR was rescued by low-temperature (27 °C) incubation for 24 h. Where indicated, forskolin, isorhamnetin, and CFTR_inh_-172 were added. (**B**) Summary of apical membrane current increases (∆Isc, mean ± S.E., *n* = 3–4). * *p* < 0.05, ** *p* < 0.01.

Patients with cystic fibrosis (CF) who have the CFTR loss of function mutation have an increased incidence of dry eye syndrome [12]. The most common mutation in CFTR in CF is ΔF508, accounting for approximately 70% of all mutations [15]. To investigate whether isorhamnetin can enhance ΔF508-CFTR activity, we measured apical membrane currents in ΔF508-CFTR expressing FRT cells. As shown in Figure 4, isorhamnetin strongly potentiated forskolin-induced ΔF508-CFTR chloride current in a dose-dependent manner. Notably, application of 30 μM isorhamnetin doubled the ΔF508-CFTR chloride current activated by 20 μM forskolin.

### 2.5. Isorhamnetin Increases Ocular Surface Chloride Secretion and Tear Volume

**Figure 5:**
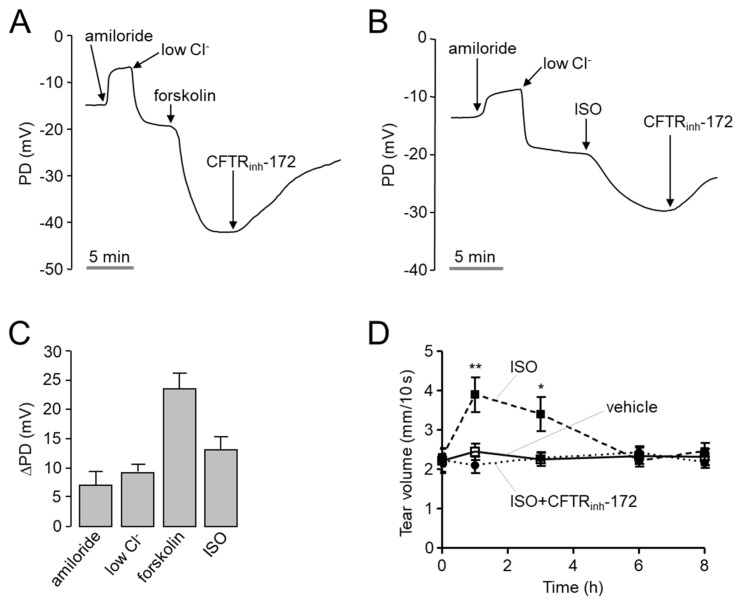
PD measurement and tear fluid volume measurement of isorhamnetin in live mice. (**A**,**B**) Representative continuous recordings of change in ocular surface open-circuit PD in response to perfusion with solutions containing indicated compounds (see Section 4). (**C**) Summary of changes in ocular surface PD (ΔPD). (**D**) Tear volume was measured at the indicated times after 2.5 μL volume of single ocular delivery of vehicle, isorhamnetin (30 µM), or isorhamnetin with CFTR_inh_-172 (20 µM) in CD-1 mice (mean ± S.E., *n* = 10). * *p* < 0.05, ** *p* < 0.01.

To investigate whether isorhamnetin can increase ocular tear volume in mice, open-circuit ocular surface potential difference (PD) was measured in CD-1 mice. As shown in Figure 5A–C, to increase the driving force for chloride secretion, ENaC was inhibited by amiloride and continuously perfused ocular surface with a solution containing low Cl- and then CFTR was stimulated by forskolin or isorhamnetin. Isorhamnetin significantly hyperpolarized ocular PD, and the hyperpolarization was inhibited by CFTR_inh_-172. To observe the effect of isorhamnetin on tear secretion in mice, tear volume was determined using phenol red thread tear test in CD-1 mice. Interestingly, isorhamnetin tear volume was significantly increased by 30 µM isorhamnetin, which induced maximal activation of CFTR in vitro, and the increased tear volume was completely suppressed by CFTR_inh_-172 (Figure 5D).

### 2.6. Isorhamnetin Ameliorates Dry Eye Disease in Scopolamine-Induced Dry Eye Mouse Model

**Figure 6:**
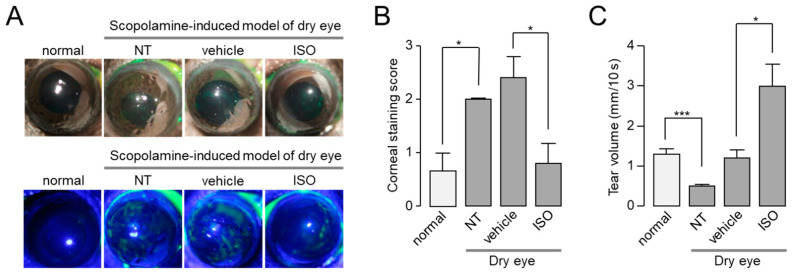
Isorhamnetin reduced ocular surface damage and increased tear fluid volume in a scopolamine-induced dry eye mouse model. (**A**) Representative photographs of mouse eyes (top) and corneal fluorescein staining images of normal and dry eye mice (bottom). Vehicle and isorhamnetin were treated three times a day for 10 days. (**B**) Degree of corneal epithelial disruption of each case measured by fluorescein staining on a five-point scale (mean ± S.E., *n* = 5). (**C**) Basal tear volume of each case measured with phenol red thread tear test (mean ± S.E., *n* = 5). NT, not treated. * *p* < 0.05, *** *p* < 0.001.

To investigate whether isorhamnetin can ameliorate dry eye disease, we observed the effect of isorhamnetin on scopolamine-induced dry eye mouse model. To examine the efficacy of isorhamnetin on ocular injury of the mice under the dry eye model, isorhamnetin or vehicle (PBS) was administered to each eye three times a day for 10 days. As shown in Figure 6, isorhamnetin significantly reduced corneal erosion and increased tear volume in the scopolamine-induced dry eye mice. Corneal erosion and reduced tear volume were significantly restored by 30 µM isorhamnetin, which induced maximal activation of CFTR in vitro (Figure 6).

### 2.7. Isorhamnetin Reduces Ocular Pro-Inflammatory Cytokines in Dry Eye Mouse

Previous studies have shown that mRNA expression levels of interleukin (IL)-1β, IL-6, IL-8, tumor necrosis factor (TNF)-α, interferon (IFN)-γ, and matrix metallopeptidase (MMP)-9 were significantly increased in tears and conjunctiva of dry eye patients compared to normal controls [16,17,18,19].

**Figure 7:**
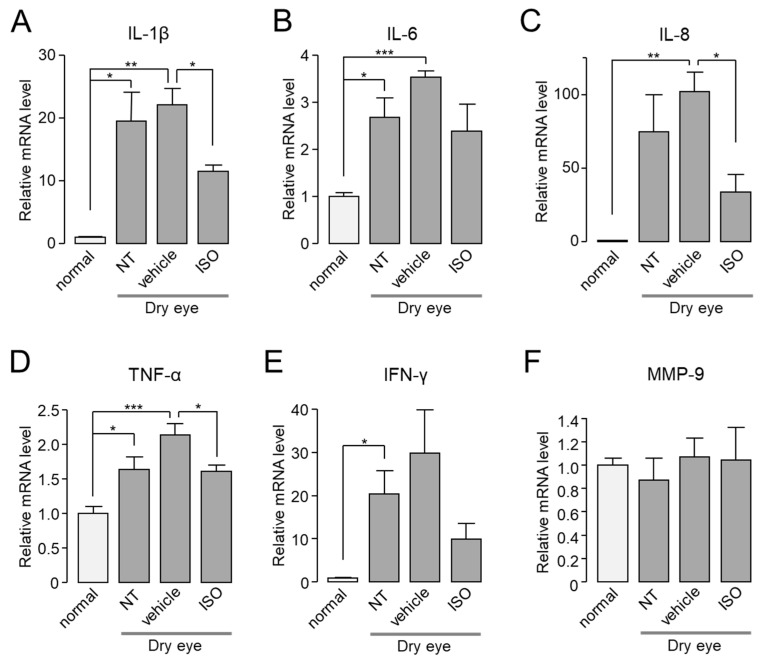
In dry eye mice, isorhamnetin reduced expression of pro-inflammatory cytokines in cornea and conjunctiva. (**A**–**F**) The mRNA expression level of IL-1β, IL-6, IL-8, TNF-α, IFN-γ, and MMP-9 in cornea and conjunctiva. Mice treated with dry eye model for 14 days, and dry eye mice treated with vehicle or isorhamnetin for 10 days while maintaining the dry eye condition (mean ± S.E., *n* = 4–5). NT, not treated. * *p* < 0.05, ** *p* < 0.01, *** *p* < 0.001.

In cornea and conjunctiva of normal or dry eye model mice, the mRNA expression levels of IL-1β, IL-6, IL-8, TNF-α, IFN-γ, and MMP-9 were investigated by real-time PCR in the presence or absence of isorhamnetin. As shown in Figure 7, the mRNA expression levels of IL-1β, IL-8, TNF-α, and IFN-γ in cornea and conjunctiva were significantly increased compared to normal mice, but the mRNA expression levels of MMP-9 were not significantly changed. Application of isorhamnetin strongly reduced the increased mRNA expression of cytokines in dry eye mice.

## 3. Discussion

Dry eye disease is a common ocular disease with a variety of symptoms such as discomfort due to inflammation of ocular surface, visual impairment, and increased osmolarity of the tear film [20]. The homeostatic maintenance of lacrimal gland and ocular surface is lost in dry eye, which, in turn, disturbs the balance of the tear film components that stabilize the tear film and protect the ocular surface [16,21]. Tear hyperosmolarity is one of the central events in the vicious circle of dry eye, leading to reduced cell volume and increased concentration of solutes. This, in turn, increases expression and production of pro-inflammatory cytokines, chemokines, and matrix metalloproteinase [16].

The inflammatory mediators such as cytokines and nuclear factor-kappa B (NF-κB) have been extensively studied in inflammatory ocular diseases such as uveitis, retinopathy, and macular edema [22,23,24], and numerous studies on the anti-inflammatory effect of natural flavonoids have been conducted over the past decades [25]. For example, myricetin has a well-established anti-inflammatory role by regulating TNF-α-stimulated production of inflammatory mediators by inhibition of NF-κB pathways [26,27], and quercetin is known for its anti-inflammatory properties primarily via downregulation of NF-κB both in vitro and in vivo [28,29]. To date, several studies have been conducted to investigate the potential use of some flavonoids, such as quercetin, in the prevention or treatment of eye diseases or disorders [30,31].

CFTR is known to be expressed in cornea and conjunctiva and provide an important pathway for fluid secretion across the ocular surface [10,32,33,34]. CFTR activity was found to be robust in a study of the electrical potential generated by the ocular surface epithelium in human subjects [13]. Thus, enhancement of CFTR Cl^-^ channel activity in the ocular epithelium by activators of CFTR can promote secretion of water to the ocular surface in patients with dry eye disease. Interestingly, topical treatment of IBMX, which stimulates CFTR by elevating the intracellular cAMP, on patients with dry eye resulted in an increase in tear secretion and decrease in tear film osmolarity [32]. In addition, patients with CF who have loss-of-function mutations in CFTR have been found to show tear film abnormalities [12,35].

In this study, we found that isorhamnetin is a bona fide activator of CFTR and induces tear secretion via CFTR activation (Figure 3 and Figure 5). Previous studies showed that isorhamnetin, a natural flavonoid, has many biological effects that are applicable to degenerative diseases, such as cardio-cerebrovascular disease, osteoarthritis, and periodontitis [36]. Isorhamnetin can prevent or reduce inflammatory reactions by regulating the activation of different signal pathways such as PI3K/AKT and NF-κB [37,38]. Isorhamnetin also downregulates the secretion of pro-inflammatory cytokines via inhibition of ERK, JNK, and NF-κB signaling pathway, and its antioxidation and anti-inflammatory effects can be helpful for the treatment of chronic inflammatory diseases that require long-term treatment [38,39]. Therefore, the dual beneficial effect of isorhamnetin on dry eye disease is expected through the antioxidant activity and the CFTR activation of the ocular surface epithelium. In addition, there are comorbidities that must be treated together with the underlying dry eye disease, such as recurrent corneal erosions [40]. Since the antioxidant effects of isorhamnetin may be beneficial for other ocular diseases, isorhamnetin should also be tested for therapeutic effects on comorbidities in the future.

Isorhamnetin potently and selectively activated both WT- and ΔF508-CFTR in a dose-dependent manner without increasing intracellular cAMP concentration (Figure 1, Figure 2 and Figure 4), and topical treatment of isorhamnetin on ocular surface effectively increased tear volume of normal CD-1 mice for several hours (Figure 5), which showed that efficient tear production and turnover of the tear film could be possible. In a scopolamine-induced dry eye mouse model, the isorhamnetin-treated group exhibited significantly increased tear volumes compared to the vehicle-treated group, and this result correlated with decrease in corneal erosion grade because isorhamnetin improved tear film stability by increasing tear production (Figure 6). Previous clinical studies on inflammatory mediators that occur in dry eye disease have shown elevated levels of IL-1β, IL-8, and TNF-α in the tear film of dry eye patients [41,42]. As shown in Figure 7, isorhamnetin significantly reduced these pro-inflammatory cytokines expressed in the cornea of the dry eye mouse model. Isorhamnetin may be a potential agent for the treatment of dry eye caused by lacrimal gland dysfunction in patients with Sjogren’s syndrome because it can induce water secretion via CFTR activation in ocular surface epithelium and has anti-inflammatory effect via its antioxidative activity.

Alternative prosecretory therapeutic strategies for dry eye disease that target ion channels on the ocular surface have been investigated. For examples, a P2Y_2_ purinergic receptor agonist, diquafosol, promotes both mucin secretion and transient fluid secretion via activation of Ca^2+^-activated Cl^−^ channels (CaCCs) in ocular surface epithelium [43,44,45]. Inhibition of ENaC activity by P-301 blocks the absorption of tear and provides driving force for water secretion in ocular surface epithelium [46]. However, a recent study on ion transport of the ocular surface epithelium in human subjects has revealed that CaCC and ENaC activities were found to be minimal compared to CFTR [13]. Therefore, CFTR activators, including isorhamnetin, which induce sustained fluid secretion in ocular surface epithelium, may be beneficial in the treatment of patients with dry eye disease.

## 4. Materials and Methods

### 4.1. Materials and Reagents

Isorhamnetin and other chemicals, unless otherwise indicated, were purchased from Sigma-Aldrich (St. Louis, MO, USA). VX-770 was purchased from Selleck Chemicals (Houston, TX, USA). CFTR_inh_-172 was synthesized as described elsewhere. The compound collections used for screening included 1000 natural products (Spectrum Collection) from MicroSource Discovery Inc. (Gaylordsville, CT, USA). Compounds were maintained as dimethyl sulfoxide stock solutions. The HCO^3−^-buffered solution contained (in mM) 120 NaCl, 5 KCl, 1 MgCl_2_, 1 CaCl_2_, 10 D-glucose, 5 HEPES, and 25 NaHCO_3_ (pH 7.4). In the half-Cl^−^ solution, 65 mM NaCl in the HCO_3_^−^-buffered solution was replaced by Na gluconate.

### 4.2. Cell Culture

Fisher rat thyroid (FRT) cells expressing human wild-type and ΔF508-CFTR with a halide sensor YFP-H148Q/I152L were generously provided by Dr. Alan Verkman (University of California, San Francisco, CA, USA) and grown in F-12 Modified Coon’s medium supplemented with 10% FBS, 2 mM glutamine, 100 units/mL penicillin, and 100 µg/mL streptomycin. T84 cells were grown in the same medium, generously provided by Dr. Min Goo Lee (Yonsei University, Seoul). CHO-K1 cells expressing human wild-type CFTR with a halide sensor YFP-H148Q/I152L and HEK-293T cells expressing human ether-a-go-go-related Gene (hERG) were grown in Dulbecco’s modified Eagle’s medium supplemented with 10% FBS, 2 mM glutamine, 100 units/mL penicillin, and 100 µg/mL streptomycin. Immortalized human corneal epithelial (CorE) cells were grown in bronchial epithelial cell growth medium (Lonza, Switzerland) with all the supplements. Immortalized human conjunctival epithelial (ConjE) cells were grown in corneal epithelial cell medium (ScienCell, CA, USA) with all the supplements.

### 4.3. Cell-Based Screening

CHO cells expressing wild-type CFTR with the halide sensor YFP-H148Q/I152L were plated in 96-well, black-walled microplates (Corning Inc., Corning, NY, USA) at a density of 2 × 10^4^ cells per well. CHO-WT-CFTR-YFP cells were incubated for 48 h at 37 °C. Assays were done using FLUO star Omega microplate reader (BMG Labtech, Ortenberg, Germany) and MARS Data Analysis Software (BMG Labtech). Briefly, each well of a 96-well plate was washed three times in PBS (200 µL/wash). Then, 100 µL PBS was added to each well. Test compounds (1 µL) were added to each well at final concentration of 25 µM. After 10 min, 96-well plates were transferred to the microplate reader preheated to 37 °C for fluorescence assay. Each well was assayed individually for CFTR-mediated I^-^ influx by recording fluorescence continuously (400 ms per point) for 2 s (baseline). Then, 100 µL of 140 mM I^-^ solution were added at 2 s and then YFP fluorescence was recorded for 14 s. Initial iodide influx rate was determined from the initial slope of fluorescence decrease, by nonlinear regression, following infusion of iodide.

### 4.4. Short-Circuit Current

Snapwell inserts containing CFTR-expressing FRT and T84 cells were mounted in Ussing chambers (Physiologic Instruments, San Diego, CA, USA). Forskolin, isorhamnetin, and CFTR_inh_-172 were added to the apical and basolateral bath solution. For FRT cells, the apical bath was filled with a half-Cl^−^ solution and the basolateral bath was filled with HCO^3-^-buffered solution to generate transepithelial Cl^−^ gradient (apical, 64 mM; basolateral, 129 mM), and the basolateral membrane was permeabilized with 250 µg/mL amphotericin B. For T84 cells, symmetrical HCO^3-^-buffered solutions were used, and ENaC was inhibited with amiloride (100 µM). All cells were bathed for a 20-min stabilization period and aerated with 95% O_2_/5% CO_2_ at 37 °C. Apical membrane current and short-circuit current were measured with an EVC4000 Multi-Channel V/I Clamp (World Precision Instruments, Sarasota, FL, USA) and recorded using PowerLab 4/35 (AD Instruments, Castle Hill, Australia). Data were collected and analyzed with ADInstruments acquisition software Labchart Pro 7 software. The sampling rate was 4 Hz.

### 4.5. Patch Clamp

Whole-cell, patch clamp recordings were performed on CFTR-expressing CHO cells. The bath solution contained (in mM) 140 NMDG-Cl, 1 CaCl_2_, 1 MgCl_2_, 10 glucose, and 10 HEPES (pH 7.4). The pipette solution contained (in mM) 130 CsCl, 0.5 EGTA, 1 MgCl_2_, 1 Tris-ATP, and 10 HEPES (pH 7.2). Pipettes were pulled from borosilicate glass and had resistances of 3–5 MΩ after fire polishing. Seal resistances were between 3 and 10 GΩ. After establishing the whole-cell configuration, CFTR was activated by forskolin and/or isorhamnetin. Whole-cell currents were elicited by applying hyperpolarizing and depolarizing voltage pulses from a holding potential of 0 mV to potentials between −80 and +80 mV in steps of 20 mV. Recordings were made at room temperature using an Axopatch-200B (Axon Instruments, San Jose, CA, USA). Currents were digitized with a Digidata 1440A converter (Axon Instruments), filtered at 5 kHz, and sampled at 1 kHz.

### 4.6. The cAMP Assay

FRT cells grown on 96-well culture plates were washed three times with PBS at 37 °C and then incubated in induction buffer from a cAMP assay kit (cAMP-Glo Assay; Promega, Madison, WI, USA) at 37 °C containing 100 μM 3-isobutyl-1-methylxanthine (IBMX) for 10 min in the absence or presence of forskolin or isorhamnetin. Cytosolic cAMP was measured using the kit according to the manufacturer’s protocol.

### 4.7. FluxOR Potassium Ion Channel Assay

HEK293 cells stably expressing human Kv11.1 (hERG) were plated in 96-well plates. After 48 h, the cells were incubated at 28 °C for 4 h to enhance membrane expression of hERG. The culture medium was replaced with 80 μL/well of FluxOR (Invitrogen) loading buffer and incubated for 1 h at 37 °C in the dark. After removal of the loading buffer, 100 µL of assay buffer were added to each well. To measure the effect of isorhamnetin on hERG channels, the cells were pretreated with isorhamnetin for 10 min. FluxOR fluorescence (excitation/emission: 490/525 nm) was recorded for 4 s before addition of 20 μL of stimulus buffer containing thallium ions, and the fluorescence was monitored. FluxOR fluorescence was recorded and analyzed using FLUOstar Omega Microplate Reader (BMG Labtech) and MARS Data Analysis Software (BMG Labtech). All buffers were prepared according to the manufacturer’s instructions.

### 4.8. Cell Proliferation Assays

Immortalized human CorE [47] and immortalized human ConjE (Innoprot, Bizkaia, Spain) cells were plated on 96-well microplates. After 24 h incubation, cells were treated with 30 μM isorhamnetin or 0.01% Triton X-100 and then they were incubated for two days. An equal amount of DMSO was added to the control. The culture medium and the compounds were changed every 12 h. To assess cell proliferation, after 48 h of incubation with the compound, the cells were reincubated with MTS for 1 h. The soluble formazan produced by cellular reduction of MTS was quantified by measuring the absorbance at 490 nm with Infinite M200 (Tecan, Grödig, Austria) microplate reader. MTS assay was done using CellTiter 96 AQueous One Solution Cell Proliferation Assay kit (Promega, Madison, WI, USA).

### 4.9. Mouse Ocular Potential Difference (PD) Measurement

CD-1 mice weighing 30 g were studied at age 6 weeks. The animal study protocols were approved by the Institutional Animal Ethics Committee of Yonsei University. Transepithelial PDs of the ocular surface were measured continuously in anesthetized mice as described [33]. Mice were anesthetized with 2,2,2-tribromoethanol (avertin, 125 mg/kg intraperitoneal; Sigma-Aldrich, St. Louis, MO, USA), with additional avertin injected during experiments to maintain anesthesia. Mice were immobilized during the experiment with a custom-built lift, and the face was positioned so that the eye could be faced upward. The eyes were kept hydrated with regular solution that had NaCl added and matched the mouse blood osmolarity at 320 mOSM flowing out from a perfusion tube connected to a reservoir with the solution in it. The tip of the perfusion tube was positioned ~1 mm above the ocular surface of the mice. Mice body temperature was kept at 37 ± 1 °C by putting a heating pad underneath the lift. For measuring the potential difference at the ocular surface, a 1-M KCl agar-bridge connected to an Ag/AgCl electrode and high-impedance digital voltmeter (6 1/2 Digits Multimeter; Picotest, Phoenix, AZ, USA) was positioned alongside the tip of the perfusion tube. The reference electrode, which consisted of a second Ag/AgCl electrode with 1-M KCl agar-bridge and 320-mOSM, saline-filled syringe needle, was inserted in the subcutaneous tissue at the back. After initial measurement of the baseline, all solutions perfused had 100 µM amiloride present. Then 20 μM forskolin, 30 μM isorhamnetin, and 20 μM CFTR_inh_-172 were added accordingly to the solution in the reservoir to treat the mouse ocular surface with each respective compound. The potential difference at the mouse ocular surface was recorded using M35XX software (Picotest) at a rate of 250 µS per point.

### 4.10. Tear Volume

Tear volume was measured using phenol red threads (Zone-Quick, Oasis Medical, Glendora, CA, USA) by placing them in the lateral canthi of normal CD-1 or dry eye model-treated BL6 mice for 10 s using forceps. Tear volume was measured using a Vernier caliper as the length of thread wetting under a microscope. For normal CD-1 mice, serial measurements were done to evaluate compound pharmacodynamics compared to the vehicle after topical application of a single, 2.5-μL drop of compound formulations containing 20 μM forskolin, 30 μM isorhamnetin, or 20 μM CFTR_inh_-172 in PBS with 0.1% DMSO.

### 4.11. Fluorescein Staining

To assess corneal epithelial disruption, 5 μL of fluorescein dye (1%) were applied to the ocular surface of mice. Photographs of the eye were taken with a digital camera. Each cornea was scored from 0 to 5 according to the Oxford scheme [48].

### 4.12. Scopolamine-Induced Dry Eye Mouse Model

Eight-week-old C57BL/6 mice were used for this experiment. Experimental dry eye was induced by subcutaneous injection of 0.5 mg/100 μL scopolamine hydrobromide (Sigma-Aldrich, St. Louis, MO, USA) three times a day with a standard desiccating environment created by placing the mice in a chamber with a continuous air flow (15 L/min) in a room at 25 °C with an ambient humidity of 12%. Fourteen days after the initiation of experimental dry eye, the mice were treated or not with 5-μL drop of ophthalmic formulation (30 μM isorhamnetin in PBS with 0.1% DMSO) or vehicle three times a day. Eight days after the initiation of the formulation treatment, scopolamine injection was stopped. Ten days after the initiation of the formulation treatment, measurement of tear volume, corneal disruption, and relative mRNA level was performed.

### 4.13. Quantitative PCR Analysis

The mRNA expressions of IL-1β, IL-6, IL-8, TNF-α, IFN-γ, and MMP-9 in cornea and conjunctiva of the dry eye model-treated mice were measured by qPCR. RNA was isolated using TRIzol reagent (Invitrogen, Carlsbad, CA, USA), and 1 μg of RNA was used to synthesize complementary DNA (cDNA) using RNA to cDNA EcoDryTM premix (TaKaRa, Shiga, Japan) according to the manufacturer’s protocol. The relative mRNA levels were measured in ViiA7 (Applied Biosystems, Foster City, CA, USA) using SYBR Green PCR Master Mix (Applied Biosystems). The primer sequences used were as follows: GAPDH, sense (5-AACGACCCCTTCATTGACCT-3) and antisense (5-ATGTTAGTGGGGTCTCGCTC-3), size of PCR product 155 base pairs; IL-1β, sense (5-ACTCATTGTGGCTGTGGAGA-3) and antisense (5-TTGTTCATCTCGGAGCCTGT-3), size of PCR product 199 base pairs; IL-6, sense (5-CTGCAAGAGACTTCCATCCAG-3) and antisense (5-AGTGGTATAGACAGGTCTGTTGG-3), size of PCR product 131 base pairs; IL-8, sense (5-CCCTGTGACACTCAAGAGCT-3) and antisense (5-CAGTAGCCTTCACCCATGGA-3), size of PCR product 190 base pairs; TNF-α, sense (5-AGCACAGAAAGCATGATCCG-3) and antisense (5-CGATCACCCCGAAGTTCAGT-3), size of PCR product 166 base pairs; IFN-γ, sense (5-TTCTTCAGCAACAGCAAGGC-3) and antisense (5- ACTCCTTTTCCGCTTCCTGA-3), size of PCR product 156 base pairs; and MMP-9, sense (5-AAAACCTCCAACCTCACGGA-3) and antisense (5-GTGGTGTTCGAATGGCCTTT-3), size of PCR product 190 base pairs. The mRNA levels of the cytokines and MMP-9 were normalized to GAPDH levels and the fold-change in gene expression was determined by using 2^-ΔΔCT^ method.

## Abbreviations

CFTR	cystic fibrosis transmembrane conductance regulator
ANO1	anoctamin-1
TMEM16A	transmembrane member 16A
TNF-α	tumor necrosis factor- α
IL	interleukin
IFN-γ	interferon- γ
ENaC	epithelial sodium channel
cAMP	cyclic adenosine monophosphate
FRT	fisher rat thyroid
CorE	corneal epithelial
ConjE	conjunctival epithelial
PD	potential difference
NF-κB	nuclear factor κ B
hERG	human ether-a-go-go-related Gene
MMP-9	matrix metallopeptidase 9
